# Meetings of Societies

**Published:** 1908-03

**Authors:** 


### MEETINGS OF SOCIETIES.

### Bristol Medico-Chirurgical Society.

*December 11th*, 1907.

Dr. Henry Waldo, President, in the Chair.

Dr. Alexander showed a case of **Cervical Scoliosis with Hemidiaphoresis.** The patient was a woman aged 61, and presented a remarkable bony swelling in the right side of her neck. She sweated on the left side of the face and body, but not on the right, and on the latter side felt undue coldness. No mydriasis was present. The speaker suggested that the deformity was the result of old tubercular disease of the cervical spine. Dr. J. O. Symes suggested that spasm of muscle (torticollis) might have caused the deformity. A skiagram showed no thickening of bone suggestive of tubercular trouble.

Dr. Alexander also read notes on a case of **Abdominal Fibroma** simulating splenomegaly. (For these notes see page 60.)

Dr. Michell Clarke read a paper on **Two cases of removal of cyst from the lateral lobe of the cerebellum,** and showed one of the two patients. The surgical treatment had been carried out by Mr. R. G. P. Lansdown.

Mr. Munro Smith read notes on **A case of ruptured middle meningeal artery—operation, recovery.** (For these notes see page 58.)

---

*January 8th*, 1908.

Dr. Henry Waldo, President, in the Chair.

Dr. P. Watson Williams showed a case of **Frontal Sinusitis** cured by operation, and another case in which the ethmoid cells had been removed and the spenoidal sinus curetted by external operation.

Dr. Chowry Muthu read a paper on **Some clinical symptoms in the early stages of Pulmonary Tuberculosis.**

Mr. A. L. Flemming read notes on a **Case of Filariasis (F. Loa).** The patient was a man of 30, who had just returned to England after being six years in German West Africa. On the journey home he had been seized with very acute pain in back. Later a swelling gradually appeared in the erector spinæ muscle, on one side of the spine. The patient became very ill, and the swelling increased and was ultimately incised by Mr. Carwardine, and a small abscess found over the last rib. The Filaria Loa was found in the blood. Recovery was rapid after the operation.

Dr. WALTER SWAYNE read a paper on the operative treatment of **Pelvic Hæmatocele,** and showed, with Dr. Walker Hall, lantern slides of chorion-epithelioma.

---

*February 12th,* 1908.

Dr. HENRY WALDO, President, in the Chair.

Dr. P. WATSON WILLIAMS showed two patients who had been operated upon for **Malignant Disease of the Nose.** The first was a man who had had a swelling in the region of one frontal sinus. An incision had been made as for frontal sinusitis, and a sarcomatous growth exposed and removed. The growth had extended in the ethmoid bone, and a large part of the inner wall of the orbit had to be removed. No recurrence was noticeable at present, three months after operation. The second patient had had a polypoid mass, involving the septum of the nose, and extending back into the naso-pharynx. A large osteo-plastic flap had been turned down from the side of the nose, and good access thereby obtained to the interior of the nose, and the growth removed. The scarring was slight.

Professor WALKER HALL gave a demonstration, with lantern illustrations, upon some points in the **Pathology of Gastric and Duodenal Ulcers.**

Mr. CARWARDINE discussed the **Surgical Treatment of Gastric and Duodenal Ulcerations,** with lantern demonstrations. He **reported** eight **cases of acute perforation** of ulcers which he had operated upon, of which the last five had been successful. Also he **reported** upon fifteen consecutive **cases of gastro-jejunostomy** without a fatality.

Dr. NIXON read a paper on **Surgical Aid in Gastric and Duodenal Ulcer.** Ten cases were analysed, and the indications for and against operation formulated.

Mr. E. W. HEY GROVES discussed the **technique of gastro-enterostomy.** In doubtful cases of pyloric thickening, the speaker laid stress upon the advantages of first performing gastro-enterostomy, and a few weeks later again examining the thickened parts. If they were non-malignant they would be found much less thickened than at the time of the first exploration. Also the speaker had found, in one case, the operation of gastro-enterostomy facilitated by drawing up the parts to be sutured through a hole made in the anterior omentum.

Dr. MICHELL CLARKE discussed the indications for surgical interference in gastric and duodenal ulcers, and their diagnosis and medical treatment.—Drs. EDGEWORTH and SWAIN continued the discussion.

J. LACY FIRTH.

J. A. NIXON, *Hon. Sec.*

